# Extracellular Amyloid Deposits in Alzheimer’s and Creutzfeldt–Jakob Disease: Similar Behavior of Different Proteins?

**DOI:** 10.3390/ijms22010007

**Published:** 2020-12-22

**Authors:** Nikol Jankovska, Tomas Olejar, Radoslav Matej

**Affiliations:** 1 Department of Pathology and Molecular Medicine, Third Faculty of Medicine, Charles University and Thomayer Hospital, 100 00 Prague, Czech Republic; tomas.olejar@seznam.cz (T.O.); radoslav.matej@ftn.cz (R.M.); 2 Department of Pathology, First Faculty of Medicine, Charles University, and General University Hospital, 100 00 Prague, Czech Republic; 3 Department of Pathology, Third Faculty of Medicine, Charles University, and University Hospital Kralovske Vinohrady, 100 00 Prague, Czech Republic

**Keywords:** Alzheimer’s disease, Creutzfeldt–Jakob disease, Gerstmann–Sträussler–Scheinker syndrome, amyloid, senile plaques, PrP plaques, plaque subtypes

## Abstract

Neurodegenerative diseases are characterized by the deposition of specific protein aggregates, both intracellularly and/or extracellularly, depending on the type of disease. The extracellular occurrence of tridimensional structures formed by amyloidogenic proteins defines Alzheimer’s disease, in which plaques are composed of amyloid β-protein, while in prionoses, the same term “amyloid” refers to the amyloid prion protein. In this review, we focused on providing a detailed didactic description and differentiation of diffuse, neuritic, and burnt-out plaques found in Alzheimer’s disease and kuru-like, florid, multicentric, and neuritic plaques in human transmissible spongiform encephalopathies, followed by a systematic classification of the morphological similarities and differences between the extracellular amyloid deposits in these disorders. Both conditions are accompanied by the extracellular deposits that share certain signs, including neuritic degeneration, suggesting a particular role for amyloid protein toxicity.

## 1. Introduction

Deposits of aggregates of particular proteins are specific hallmarks of a wide range of neurodegenerative diseases [1]. Aggregates of misfolded proteins with altered degradation can be located intracellularly and/or extracellularly. The most important primary intracellular proteins include:

1. Hyperphosphorylated protein tau in Alzheimer’s disease (AD) [2], tauopathies including frontotemporal lobar degenerations with tau pathology (FTLD-tau) [3];
2. Alpha-synuclein in Lewy bodies in Parkinson disease (PD) and dementia with cortical Lewy bodies (DLB) or in oligodendroglial inclusions in multiple systemic atrophy (MSA);
3. Phosphorylated TDP-43 in frontotemporal lobar degeneration with TDP-43-positive inclusions (FTLD-TDP) [4];
4. Ubiquitin in frontotemporal lobar degeneration with inclusions positive for ubiquitin-proteasome system markers (FTLD-UPS) [4,5];
5. Fused in sarcoma (FUS) inclusions in FTDL-FUS [6].

Primary extracellular protein aggregates, in optical microscopy called “plaques,” can be observed in cortical locations in:

1. AD [7];
2. Prion diseases (Creutzfeldt–Jakob disease (CJD), Gerstmann–Sträussler–Scheinker syndrome (GSS), fatal familial insomnia (FFI), and kuru) [8].

In all of these diseases, the term cerebral amyloidosis is widely used referring to insoluble fibrillar structures with a predominant beta-sheet conformation detectable by Congo red and thioflavin S binding [9]. These pathologic units are known to form from insoluble fibrils, giving rise to tridimensional aggregates called plaques that may exhibit different features depending on subtype.

The aim of our review is to compare and highlight similarities and differences between the two types of extracellular deposits, i.e., Aβ in AD and amyloid prion protein in prionoses, while simultaneously synthesizing the available information for didactic purposes.

## 2. Alzheimer’s Disease

Alzheimer’s disease (AD) is a progressive neurodegenerative disease and is the most common form of dementia [10]. The prevalence in those over 65 years is reported to be 3%, and in those over 85 years, it is about 32% [11]; therefore, as the human population ages, the total number of AD patients will increase. The neuropathological diagnostic hallmarks fundamental to an AD are extracellular Aβ plaques and intracellular neurofibrillary tangles (NFTs), both of which are neuropathologically defined using the National Institute on Aging–Alzheimer’s Association (NIA-AA) consensus scheme [12,13]. Extracellular amyloid deposits are evaluated according to Thal’s criteria, in which the phase is based on the brain areas manifesting Aβ plaques, the extent of intracellular neurofibrillary tangles, according to Braak staging [10,13], and semiquantitatively estimated density of neocortical neuritic plaques as recommended by the Consortium to Establish a Registry for Alzheimer’s Disease (CERAD) [14]. From all Aβ species, Aβ oligomers are considered to be the most toxic and most likely to lead to neuronal dysfunction and degeneration. Moreover, Aβ fibrils share experimental properties of transmissibility with prion proteins, and more research is needed into the “prionoid” or “prion-like” biochemical phenomena of all amyloidogenic peptides [15]. Hence, oligomeric Aβ concentrations impact cognitive impairment more than concentrations of Aβ monomers or plaques themselves [16], although the precise role of Aβ in AD pathophysiology is still not fully understood. Nevertheless, in AD, the decline in cognitive function is most closely related to the occurrence of NFTs than of Aβ deposits [17].

### 2.1. Background of Aβ Plaque Formation

Amyloid precursor protein (APP), a transmembrane protein existing in several isoforms [18], is amply expressed in brain tissue [19], and it plays a role in neuroprotection and homeostasis [20]. Additionally, APP is able to bind heparin and metals, mainly zinc [20] and copper [21]. When added exogenously, APP protects cell cultures from Aβ toxicity [22]. Through proteolysis, using β-secretase and γ-secretase [23], it creates Aβ polypeptides that are 38–43 amino acids long [24]. The whole pathway of APP processing involves the initial cleavage, by β-secretase, to clip off the N-terminal fragment (sAPPβ). Then γ-secretase cleaves the residual APP C-terminal fragment creating Aβ, and the amyloid intracellular domain (AICD) is formed. According to studies on primary neuronal cultures, cell viability is significantly reduced when β- or γ-secretase is inhibited or during Aβ immunodepletion [25].

The 42-amino-acid-long Aβ (Aβ42) is the main component of senile plaques, whereas Aβ 40, the more abundant product of APP processing [26], and which is less prone to aggregation, is common around blood vessels [27,28]—especially leptomeningeal, and small or medium-sized cortical arteries, arterioles, and capillaries [29]. While Aβ40 is described as a “closed” tetramer that is relatively resistant to the addition of additional Aβ40 units, Aβ42 is a more “open” tetramer with a tendency to generate hexameric and subsequently more stable dodecameric structures [30,31]. As mentioned above, the Aβ42 oligomers are considered to be the most toxic and causative in the development of AD [32,33].

### 2.2. Theory—Amyloid Cascade Hypothesis

In 1992, Hardy and Higgins [34] articulated the theory that the deposition of Aβ protein, the main component of plaques, was the causative agent of Alzheimer’s pathology and that neurofibrillary tangles, cell loss, vascular damage, and dementia follow as a direct result of this deposition. The theory is supported by:

1. An occurrence of familial Alzheimer’s disease (fAD) in patients carrying an autosomal dominant mutation in genes encoding APP.
2. A higher fAD incidence was seen in families carrying the presenilin 1 (PSEN1) and presenilin 2 (PSEN2) mutations, which are the catalytic components of γ-secretase [35]. Most mutations in APP or PSEN1/PSEN2 alter APP proteolysis and result in increased production of the longer form of Aβ (i.e., Aβ42) [36].
3. Early-onset Alzheimer disease (EOAD) is manifested in patients with Down syndrome. The trisomy of chromosome 21, on which the gene for APP is located, logically leads to a triplicate of the *APP* gene. Many patients suffering from Down syndrome develop AD at an early age. The presence of Aβ plaques in these patients is often described in childhood [37], and the formation of neurofibrillary tangles occurs at about the age of 40 [38]. Thence, Down syndrome is considered to be the most significant genetic risk factor for the development of AD [39].

Although this theory dominates the field of AD research, it is not universally accepted [40,41,42,43], although the importance of the role of tau protein in the pathogenesis of AD and severity of cognitive decline has been demonstrated [36].

It is sometimes questioned for the following reasons:

1. There are patients having numerous plaques (or even fulfilling the neuropathological criteria for AD) but have no clinical signs of cognitive impairment [44].
2. Conversely, some mouse models of AD show memory deficits before the development of Aβ plaques [45].
3. While senile plaques appear first in the frontal cortex and then spread beyond the cerebral cortex to the hippocampus and beyond, neurofibrillary tangles initially develop in the limbic system [36]. To this day, the mutual relationship between these two neuropathological hallmarks is not fully understood.

The precise role of Aβ and tau protein in the pathophysiology of AD is still waiting for an explanation.

### 2.3. Morphological Classification of Senile Plaques (SP)

**Figure 1:**
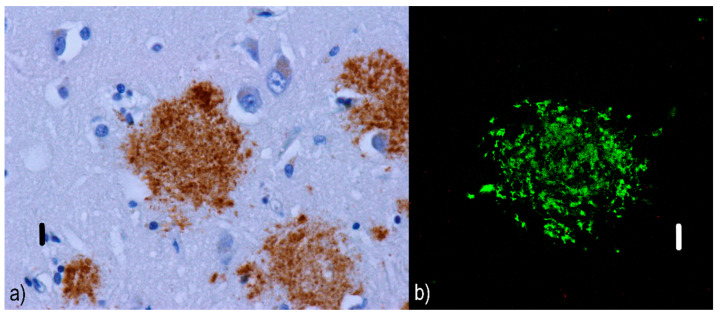
Diffuse plaques: (**a**) immunofluorescence visualization of diffuse Aβ plaques in an Alzheimer’s disease (AD) patient. Compared to non-cored plaques, diffuse ones have less defined contours; they seem lighter and less dense. Primary antibodies: anti-beta amyloid rabbit immunoglobulin G (IgG). The original magnification was 400×. The scale bar indicates a length of 10 micrometers. (**b**) Utilizing immunofluorescence confocal microscopy, the absence of tau-positive dystrophic neurites (red) in diffuse Aβ (green) plaques is evident. Primary antibodies: Anti-beta amyloid rabbit IgG and AT8 (murine anti-hyperphosphorylated protein tau). The secondary antibody was conjugated with either Alexa^®^488 (anti-rabbit IgG, green) or Alexa^®^568 (anti-mouse IgG, red). The scale bar indicates a length of 10 micrometers. The sample comes from a 92-year-old male whose neuropathological findings were a fully developed late form of Alzheimer’s disease in the neocortical phase (Braak VI, Consortium to Establish a Registry for Alzheimer’s Disease (CERAD) C, Thal 6) with local mild cerebral amyloid angiopathy (CAA Vonsattel grade 1). According to the revised “ABC” of the National Institute on Aging (NIA) classification, the changes associated with AD are at a “high” level (A3B3C3). This plaque was photographed in the subiculum, where diffuse and non-cored neuritic plaque were predominant.

**Figure 2:**
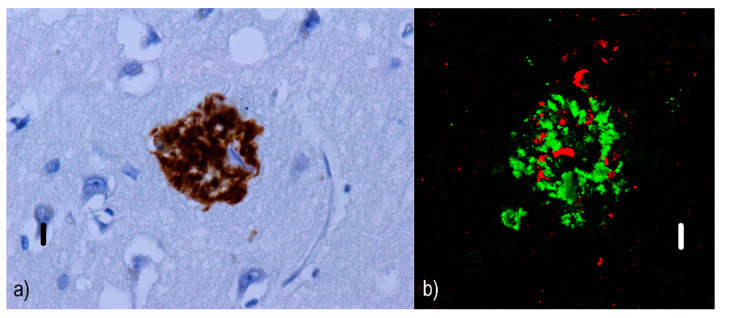
Non-cored neuritic plaques: (**a**) immunofluorescence visualization of non-cored Aβ plaque in an AD patient. These plaques are denser and more clearly bordered than diffuse ones. Primary antibodies: anti-beta amyloid rabbit IgG. The original magnification was 400×. The scale bar indicates a length of 10 micrometers. (**b**) Simultaneous imaging with a confocal microscope allowed us to display the presence of Aβ structures (green) as well as tau-positive dystrophic neurites (red) in the vicinity, which are a characteristic component of both types of neuritic plaques (either non-cored or cored). Note that some of the dystrophic neurites are dilated. Primary antibodies: anti-beta amyloid rabbit IgG and AT8 (murine anti-hyperphosphorylated protein tau). The secondary antibody was conjugated with either Alexa^®^488 (anti-rabbit IgG, green) or Alexa^®^568 (anti-mouse IgG, red). The scale bar indicates a length of 10 micrometers. The sample comes from a 67-year-old female patient with a fully developed early form of Alzheimer’s disease in the neocortical stage (Braak VI, CERAD C) with marked amyloid angiopathy (CAA Vonsattel grade 3). The changes associated with AD are at a “high” level (A3B3C3) according to the revised “ABC” classification of the NIA. This plaque comes from the amygdala region, where non-cored and cored neuritic plaques prevail in this case.

**Figure 3:**
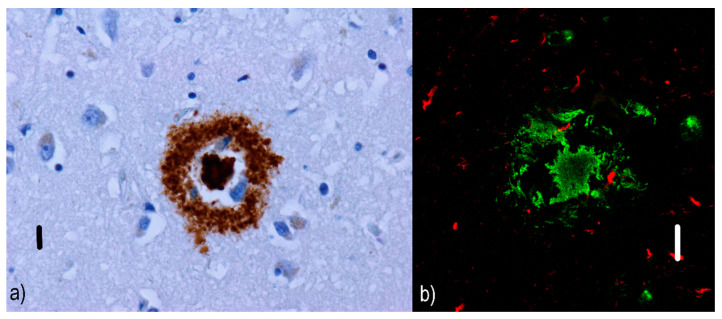
Cored neuritic plaques: (**a**) immunofluorescence visualization of cored Aβ plaque in an AD patient. The dense Aβ core is encircled by fibrillar Aβ deposits, which are clearly visible in cored neuritic plaques. Primary antibodies: anti-beta amyloid rabbit IgG. The original magnification was 400×. The scale bar indicates a length of 10 micrometers. (**b**) Simultaneous imaging with a confocal fluorescent laser scanning microscope shows the presence of an Aβ core with fibrillar Aβ structures (green) in the vicinity as well as a few tau-positive dystrophic neurites (red). Primary antibodies: Anti-beta amyloid rabbit IgG and AT8 (murine anti-hyperphosphorylated protein tau). The secondary antibody was conjugated with either Alexa^®^488 (anti-rabbit IgG, green) or Alexa^®^568 (anti-mouse IgG, red). The scale bar indicates a length of 10 micrometers. The images are from a male 67-year-old patient with EOAD and come from the cornu ammonis, but similar findings were present in all areas of the hippocampal formation and adjacent para-hippocampal and entorhinal cortex. Neuropathological diagnosis: Fully developed early-onset form of Alzheimer’s disease in the neocortical stage (Braak VI, CERAD C) with marked amyloid angiopathy (CAA Vonsattel grade 3). According to the revised “ABC” of the NIA classification, the changes associated with AD are at a “high” level (A3B3C3).

**Figure 4:**
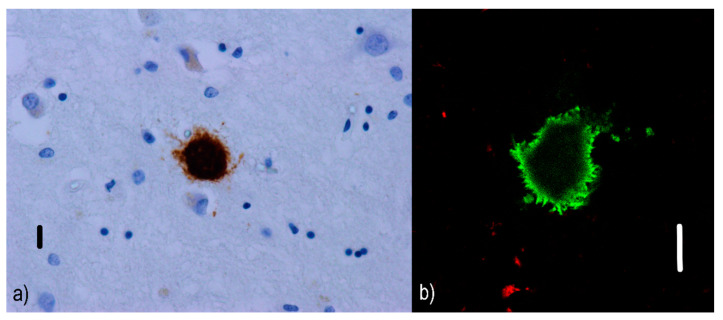
Burnt-out plaques: (**a**) immunofluorescence visualization of a burnt-out Aβ plaque (the dense core remains) in an AD patient. Primary antibodies: anti-beta amyloid rabbit IgG. The original magnification was 400×. The scale bar indicates a length of 10 micrometers. (**b**) Imaging of the dense Aβ nucleus (green) lacking surrounding components using a confocal microscope. Primary antibodies: Anti-beta amyloid rabbit IgG and AT8 (murine anti-hyperphosphorylated protein tau). The secondary antibody was conjugated with either Alexa^®^488 (anti-rabbit IgG, green) or Alexa^®^568 (anti-mouse IgG, red). The scale bar indicates a length of 10 micrometers. This image comes from the amygdala of an 83-year-old female with a late variant of AD in the neocortical stage (Braak V, CERAD C). The changes associated with AD are at a “high” level (A3B3C3) according to the revised “ABC” classification of the NIA. Burnt-out and cored neuritic plaques were predominant in this area of the patient’s brain.

Amyloid/senile plaques are extracellular deposits of Aβ that are abundant in the cortex of AD patients [46], which, on average, are about 50 µm in diameter [47]. They can be divided into three subcategories (see summary in Table 1):

1. Diffuse/pre-amyloid plaques (Figure 1) that are predominantly 10–20 µm [48] amorphous amyloid deposits with ill-defined contours [46] and lacking dystrophic neurites [49]. Diffuse plaques are not associated with a glial response [50] or synaptic loss; hence, they are not sufficient for a neuropathological diagnosis of AD. Moreover, diffuse plaques are commonly found in the elderly without signs of cognitive decline [51]. They are evident with silver staining, but invisible with Congo red [52] or thioflavin [53].
2. Two subtypes of neuritic plaques can be distinguished.
  - Non-cored/primitive/immature neuritic plaques (see Figure 2) are oval or spherical structures containing Aβ and altered neurites, 20–60 µm in diameter and lacking a dense Aβ region in the central part [54]; they are also associated with astrocytic and glial responses. They are reported to occur in older AD patients [55]. Similar to diffuse plaques, they do not stain with Congo red since they do not contain Aβ in the beta-sheet conformation [56].
  - Cored/classic/dense/mature/focal neuritic plaques (Figure 3) are 20–60 µm [53] compact cores encircled by fibrillar Aβ deposits [51]. Tau-positive dystrophic neurites [57], reactive astrocytes, and activated microglia [58,59] are found in the vicinity. Due to its relation to neuronal loss and its association with cognitive decline [60,61], these plaques are a basis for an AD diagnosis [62]. They can be visualized with silver staining [63], Congo red [64], and thioflavin [57].
3. Compact/burnt-out plaques (Figure 4) are 5–15 µm [48] in diameter, composed of a dense core that lacks a surrounding neuritic component [65].

It is not entirely clear whether non-cored neuritic plaques progress into cored and then to burnt-out plaques. In addition, it is also not known whether diffuse plaques are a common part of aging or the initial stage of neuritic plaque maturation [66].

### 2.4. Dystrophic Neurites as a Component of Aβ Plaques

Dystrophic neurites in plaques may differ morphologically and immunohistochemically. Type I is described as elongated in shape, whereas type II is dilated, bulbous, or globular [67]. Certain levels of dilated, ubiquitin-positive neurites have been previously reported in AD patients, although usually without information regarding the exact brain location [68]. Based on our observations, bulbous neuritic changes are prominent mainly in archicortical structures [69].

### 2.5. The Molecular Composition of Aβ Plaques

1. The results of immunohistochemical examinations showed that diffuse/pre-amyloid plaques contain Aβ42 and other APP fragments lacking the C-terminus [70], apolipoprotein E [71], α1-antichymotrypsin [72], complement proteins [73,74], and heparan sulfate proteoglycan (HSPG) [75].
2. According to Armstrong [70], non-cored/primitive/immature neuritic plaques additionally contain both free and conjugated ubiquitin, paired helical filament antigen (PHF-antigen), phosphorylated tau protein, and numerous immunoreactive neurites.
3. Cored/classic/dense/mature/focal neuritic plaques consist of an Aβ42 core and a ring of alpha-synuclein. In addition to Aβ42, they contain Aβ40, complement proteins, immunoglobulins, and apolipoproteins D [76] and E. Due to the secondary binding to Aβ, zinc, copper [77], or aluminum [78] may also be part of the core, with aluminum having the lowest affinity [79]. Chromogranin, interleukine-6 [80], or catecholamine-positive neurites are constituents of the ring.

### 2.6. Laminar Distribution of Aβ Plaques

The internal pyramidal layer (layer V) and the external pyramidal layer (layer III) are the most affected [81]. The reason may be that APP mRNA is expressed in huge amounts by the pyramidal neurons in the internal and external pyramidal layer [82]. The degeneration of these neurons may increase APP secretion and, consequently, Aβ plaque formation [83]. Interestingly, no differences in plaque stratification were observed between patients with early-onset fAD, late-onset fAD, or sporadic AD; even the *Apo E* genotype does not appear to affect the morphology and distribution of Aβ plaques. Moreover, no differences in plaque density between the sporadic and familial AD variants have been observed [84].

## 3. Prion Diseases

Prion diseases are transmissible, progressive, and in all cases, fatal neurodegenerative disorders associated with an aggregation of misfolded prion protein [85]. Human transmissible spongiform encephalopathies include Creutzfeldt–Jakob disease (CJD), Gerstmann–Sträussler–Scheinker syndrome (GSS), kuru, and the extremely rare fatal familial insomnia (FFI) [86]. In general, the neuropathological hallmarks of transmissible spongiform encephalopathies (TSEs) are spongiform changes, astrogliosis, and neuronal loss [87]. The toxicity of the scrapie isoform of the prion protein (PrPSc) remains controversial inasmuch as studies report different results. According to some studies, PrPSc oligomers are the most toxic form [88]; however, others state that PrPSc is not directly toxic to neurons; instead, it is the lack of the physiological cellular prion protein (PrPC) variant that leads to neuronal death [89].

Extracellular deposits and PrPSc plaques are structures visible with hematoxylin-eosin staining, while plaque-like structures can only be visualized by using immunohistochemical methods. [90] Plaques are present in 10–15% of Creutzfeldt–Jakob disease cases, 50–75% of [91,92] kuru patients, and 100% of patients suffering from Gerstmann–Sträussler–Scheinker syndrome. These amyloid plaques consist of PrP (see summary in Table 2) [93].

### 3.1. Molecular Background and the Composition of PrP-Amyloid Plaques

Cellular prion protein (PrPC) is a glycolipid-anchored cell membrane sialoglycoprotein localized on presynaptic membranes. PrPC appears to have neuroprotective [94] and pro-myelinating [95] functions; it participates in myelin maintenance, neurotransmission, zinc and copper transport, and calcium homeostasis [96,97,98]. It also seems to promote greater neuronal resistance after ischemic cerebral insult in laboratory rodent models [99,100]. An explanation for its numerous functions may be the ability of PrPC to interact with a variety of membrane proteins [98]. PrP is able to aggregate into amyloid [101] 8–10 nm long [102] and act as a receptor for Aβ [103,104]. According to recent research, the expression of PrPC is controlled by AICD [105], which was mentioned above as a product generated by γ-secretase cleavage in AD.

Clusterin often co-localizes in PrPSc plaques [101] and is able to bind Aβ, immunoglobulins, complement proteins, and lipids [106,107,108,109,110,111,112,113]. Moreover, prion protein also acts as a receptor for laminin, a glycoprotein mainly found in basement membranes [114].

### 3.2. Kuru

Kuru was the first human prionosis to be discovered and is defined as a neurodegenerative, non-inflammatory infectious disease [115,116]. Although the neurological symptoms are very similar in all patients, the neuropathological findings differ widely [117]. Shrunken neurons with dispersed Nissl bodies and intracytoplasmic vacuoles may be present, as well as vacuolated striatal neurons and cerebellar Purkinje cells [91]. A neuropathological feature may be a spongiform transformation [118] (mostly described as subtle) and neuronophagy affecting predominantly the deeper cortical layers but completely sparing hippocampal neurons. Microglial and astroglial proliferation can also be seen [117]. The most typical feature is amyloid “kuru” plaques, which are present in 50–75% [91,92] of examined brains. Immunohistochemistry has verified that the scrapie isoform of the prion protein shows synaptic and perineuronal positivity [119,120].

### 3.3. Creutzfeldt–Jakob Disease

Creutzfeldt–Jakob disease (CJD) is a transmissible and rapidly progressive [121] degenerative disease of the central nervous system caused by an accumulation of pathologically conformed PrP, [122] and the most common of the human prion diseases [123]. The neuropathological definition of CJD is spongiform encephalopathy in the cerebral and/or cerebellar cortex and/or the subcortical grey matter. Variations include encephalopathy with PrP immunoreactivity (plaque and/or diffuse synaptic and/or patchy/perivacuolar types) [124]. Four types, i.e., sporadic (sCJD), familial (fCJD), iatrogenic (iCJD) [125], and variant CJD (vCJD) [126], are distinguishable relative to their different etiologies [127]. The first mentioned, i.e., the sporadic type, is contingent on the accidental conversion of normal PrP to a pathological form and accounts for about 85% of CJD cases [128]. The genetic variant is conditioned by the detection of an inherited mutation in the prion protein gene (*PRNP*), which accounts for 10–15% of cases [129].

The other two types can be placed into the category of acquired CJD, i.e., the CJD variant that occurs after consumption of beef from cattle affected by bovine spongiform encephalopathy (BSE). The iatrogenic variant arises during medical or surgical procedures during which pathologically conformed prions are inadvertently transferred (e.g., during neurosurgical interventions, dura mater or corneal grafting, deep electrode insertions, or extraction of human pituitary hormones) [130]. Neuropathological changes include spongiform transformation, neuronal loss, astrocytosis, and the formation of PrP-amyloid plaques in the gray matter. The expression of neuropathological features varies significantly between individuals [131]. Importantly, amyloid plaques do not occur in all patients with sCJD, only accounting for approximately 10–15% of cases [124,132,133,134].

Different subtypes of sCJD are distinguishable, according to different polymorphisms at codon 129 (i.e., methionine or valine homozygosity (MM or VV, respectively) or methionine and valine (MV) heterozygosity) of the *PRNP* and the type of proteinase K-resistant prion protein fragments (PrP), using a western blot examination [135].

Character and Typical Location of PrP Deposits According to the MV Polymorphism

- MM1 subtype: synaptic and perivacuolar positivity, although cases with plaques in the white matter are so rarely encountered, we will not mention them in more detail [136].
- MM2
  - Cortical subtype: perivacuolar positivity in all cortical layers;
  - Thalamic subtype: fewer plaques (which are usually described as coarse) [137]
- MV1 subtype: synaptic and perivacuolar positivity;
- MV2 subtype: distinctive “kuru-like” plaques in the cerebellum and perineuronal positivity in the cerebral cortex;
- VV1 subtype: characterized by punctate synaptic positivity in the cerebral cortex;
- VV2 subtype: perineuronal, with numerous plaque-like areas and some synaptic PrP positivity in the cerebral cortex [138].

As mentioned above, plaques are a neuropathological hallmark, but only for the MV2 subtype, where “kuru-like” plaques are found in the granular and molecular layers of the cerebellum [139]. Sometimes the Purkinje cell layer is also described as having an abundance of plaques [140]. They are sometimes found in the subcortical gray matter but seldom in the cerebral cortex [141]. Rarely, individuals with the MM type 1 polymorphism have plaques in the white matter. In these cases, significantly longer survivals have been reported (around 24 months) [142]. These “kuru-like” plaques are characterized by a hyaline eosinophilic core with a pale halo, both visible with hematoxylin-eosin staining.

### 3.4. Gerstmann–Sträussler–Scheinker Syndrome

Gerstmann–Sträussler–Scheinker syndrome (GSS) is defined as a slowly progressive hereditary autosomal dominant neurodegenerative disease [143] or encephalo(myelo)pathy with multicentric PrP plaques [124] localized in the cerebral and cerebellar cortex and the basal ganglia [144,145]. Clinically, ataxia and progressive dementia are distinctive [146]. GSS was the first human prion disease to be associated with a *PRNP* mutation. To date, point mutations at codons 102, 105, 117, 131, 145, 187, 198, 202, 212, 217, and 232 have been reported [143]. Some families carry octapeptide repeat insertions (OPRI), families having four [147], five [148], six [149,150], seven [151], eight [152], and nine [153] multiples of the 24 base pairs between codons 51 and 91 in the *PRNP* gene have been reported. In patients with 4 to 7 multiples, elongated PrP deposits are usually described, while in those having 8 or 9 OPRI, kuru-like or multicentric plaques have been found [154]. According to some studies, clinical and neuropathological variability is further affected by MV polymorphisms at codon 129; however, other researchers have failed to find any significant differences between homozygotes and heterozygotes [155].

Using silver staining methods, amyloid plaques in prion diseases can mimic burnt-out Aβ42 plaques. Nevertheless, unlike Aβ42 plaques, these PrP plaques can be clearly seen with hematoxylin-eosin staining. After proteinase pre-treatment, the presence of PrPSc can be confirmed by using specific immunohistochemistry. While PrPSc in GSS is partially sensitive to the effects of proteinase [73].

### 3.5. Summary of Morphological Types of PrP Plaques in TSEs

**Figure 5:**
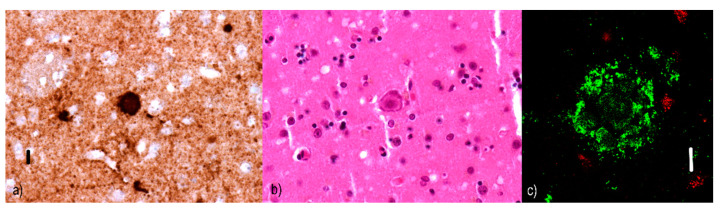
“Kuru-like” plaques: (**a**,**b**) Comparison of immunohistochemical images of “kuru-like” plaque with its correlate using hematoxylin-eosin staining. Primary antibodies: anti-prion protein (anti-PrP) rabbit IgG. The original magnification was 400×. The scale bar indicates a length of 10 micrometers. (**c**) The dense PrP nucleus and thin amyloid bundles in the periphery (green) of the “kuru-like” plaque were visualized using a confocal microscope. Tau-positive dystrophic neurites (red) are also included. Primary antibodies: anti-PrP rabbit IgG and AT8 (murine anti-hyperphosphorylated protein tau). The secondary antibody was conjugated with either Alexa^®^488 (anti-rabbit IgG, green) or Alexa^®^568 (anti-mouse IgG, red). The scale bar indicates a length of 10 micrometers. The samples come from a 74-year-old woman suffering from CJD and come from the hippocampal formation, which contained numerous plaques; patchy synaptic and peri-vascular positivity were also present. The polymorphism at codon 129 was MV.

**Figure 6:**
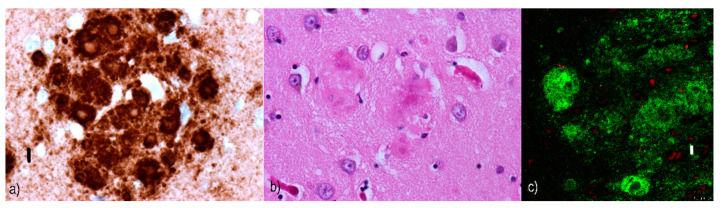
Multicentric plaques: (**a**,**b**) comparison of immunohistochemical staining of “multicentric” plaques distinctive for Gerstmann–Sträussler–Scheinker syndrome (GSS) with its correlate using hematoxylin-eosin staining. Primary antibodies: anti-PrP rabbit IgG. The original magnification was 400×. The scale bar indicates a length of 10 micrometers. (**c**) Numerous PrP plaques merged in a multicentric plaque (green), including tau-positive dystrophic neurites (red), visualized using confocal microscopy. Primary antibodies: Anti-PrP rabbit IgG and AT8 (murine anti-hyperphosphorylated protein tau). The secondary antibody was conjugated with either Alexa^®^488 (anti-rabbit IgG, green) or Alexa^®^568 (anti-mouse IgG, red). The scale bar indicates a length of 10 micrometers. All these images come from the occipital cortical area of a 69-year-old female with GSS in comorbidity with primary age-related tauopathy (PART). A causative point mutation in the *PRNP* gene was also detected (P102L).

**Figure 7:**
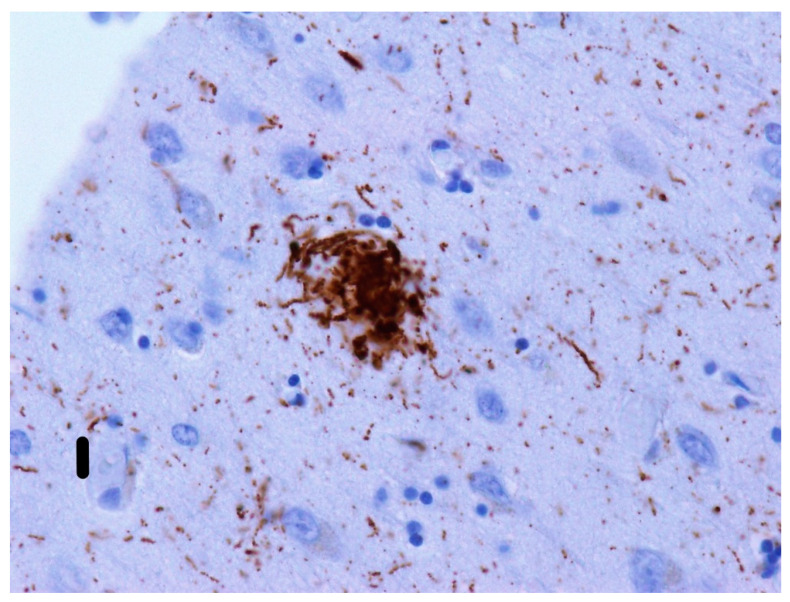
Neuritic plaques: purely neuritic plaque formed by only tau-positive neurites (stained immunohistochemically). These types of plaques are rarely found. In the above-mentioned 69 years old female patient (Figure 6) with GSS/PART, only a single neuritic plaque was detected. It was present in a section from the temporal cortex and found using immunohistochemical methods, but not in other sections examined using confocal microscopy. Primary antibodies: AT8 (murine anti-hyperphosphorylated protein tau). The original magnification was 400×. The scale bar indicates a length of 10 micrometers.

1. Unicentric/“kuru”/”kuru-like”/stellate plaques (Figure 5) are up to 30 µm [132] deposits consisting of a dense star-shaped core with thin amyloid bundles radiating into the periphery [156]. In kuru disease, the average plaque size is reported to be between 20–60 µm [117]. These plaques are surrounded by astrocytic processes that have been extensively invaded by microglia [157], although dystrophic neurites are unusual [156]. However, some studies report tau-immunoreactivity around “kuru-like” plaques [158]. “Kuru-like” plaques are present in 10–15% of sCJD patients [156], all of whom carry the MV2 polymorphism at codon 129 [138]. In CJD cases, they occur mostly in the molecular layer of the cerebellum and the Purkinje cell layer [140]. For kuru disease, typical locations include the granular cell layer of the cerebellum, the basal ganglia, thalamus, and cerebral cortex [158]. These plaques are visible with hematoxylin-eosin staining [90], which distinguishes them from plaque-like structures.
2. Daisy/florid plaques measure up to 200 µm [132] and consist of a PrP-amyloid core surrounded by a “ring” of spongiform changes. Radiating fibrils are organized into thick structures, which stand in contrast to the thin structures seen in “kuru-like” plaques [158]. There are numerous tau-immunoreactive dystrophic neurites in the vicinity that distinguish them from “kuru-like” plaques. Moreover, Hirano bodies (in the processes around florid plaques) can sometimes also be found [158]. These plaques are characteristic [159], although not specific [160] for vCJD. They can occur anywhere in the cerebral cortex but are generally found occipitally and in the cerebellar molecular layer [161]. Florid plaques are visible when stained with hematoxylin-eosin [162].
3. Multicentric plaques (see Figure 6) are formations up to 1500 µm [132] and are composed of many cores of different sizes that have merged. Unlike “kuru-like” plaques, they are characterized by the presence of dystrophic neurites [140]. Dystrophic neurites sometimes contain paired helical filaments (PHFs) identical to those seen in the dystrophic neurites of AD patients [163]. These larger cores tend to be surrounded by smaller amyloid deposits [156]. Like the previously mentioned plaques, they can be observed with hematoxylin-eosin staining [164].
4. Pure neuritic plaques (Figure 7) are the rarest type of plaques among prion diseases. Neuritic plaques consist only of clusters of dystrophic neurites with various morphologies and lack an amyloid component. They are surrounded by astrocytic processes in the immediate vicinity [156].

Both types of plaques are formed by amyloid structures—in AD by Aβ and in TSEs by prion amyloid. We tried to highlight the similarities and differences in their occurrence and behavior.

Similarities:

1. All of these diseases are based on a perturbance of proteins having physiological functions on the neuritic membrane to which they are anchored. Physiologically, they have a neuroprotective function and are able to interact with a number of other agents.
2. They are also similar to each other in the resistance of these extracellular aggregates to degradation by endogenous proteases.
3. In both AD and TSEs, extracellular aggregates may form not only compact structures such as plaques but also diffuse extracellular deposits.
4. For all mentioned diseases, extracellular deposits are mainly found in the cortical areas or in the central grey matter. Their presence in white matter is possible but exceedingly rare in TSEs and absolutely unheard of in Alzheimer’s disease.
5. When forming plaques, they usually contain dystrophic neurites with similar immunohistochemical characteristics in both AD and TSEs. The neuritic morphology can vary from case to case.
6. The most toxic and neuronal death-inducing forms are oligomeric assemblies of both Aβ and PrP.

Dissimilarities:

1. While Aβ has thread-like morphology, PrP tends to be more lumpy or globular.
2. In AD, plaques probably mature, i.e., the individual types probably transform from one to the next. Nothing like “plaque maturation” has been recorded in prionoses.
3. Especially in GSS, plaque fusion and the formation of multicentric structures are distinctive. No similar trends are seen in AD.
4. For prionoses, different appearances, locations, and frequencies of extracellular aggregates are reported depending on the form and subtype. In AD, neuropathological differences between early and late-onset or sporadic and familial variants have never been described.
5. In TSEs, PrP deposits may be found intracellularly in some patients, while the occurrence of Aβ is strictly extracellular.
6. In AD and prionoses, there is a different trend relative to the spread of deposits within the brain. In AD, we distinguish five phases, with phase 1 being characterized by the presence of Aβ deposits limited to neocortical areas. During phase 2, the archicortical and paleocortical (together called allocortical) regions are affected. This is followed by a spread to the striatum and subcortical nuclei in general during phase 3. Brainstem involvement defines phase 4, and the involvement of the cerebellum defines phase 5 [165]. In prionoses, no stages are distinguishable since there is no characteristic spreading pattern over time.

## 4. Conclusions

To our best knowledge, this is the first systematic classification of the morphological similarities and differences between the extracellular amyloid deposits in AD and CJD. The work also clearly demonstrates the broad spectrum of these specific neuropathological entities. Better clarification of the processes of extracellular aggregate formation of different amyloidogenic proteins may be helpful for understanding the development of individual neurodegenerations and, thus, could be a useful tool for the development of effective and precise biological treatments for these progressive and fatal disorders.

## Figures and Tables

**Table 1:** Summary of Aβ plaque types in AD.

Amyloid/Senile Plaques			
- Extracellular deposits of amyloid-β abundant in the cortex of AD patients [46] - Diameter ~50 µm [47]			
**Diffuse/pre-amyloid**	**Neuritic**		**Compact/burnt-out**
- predominantly 10–20 µm - amorphous Aβ deposits with ill-defined contours [46] - lacking dystrophic neurites [49] - not associated with glial response [50] or synaptic loss [51] - not sufficient for the neuropathological diagnosis of AD - commonly found in brains of elderly without cognitive decline [46] - evident using silver stain - invisible with Congo red [52] or thioflavin [53]	**Non-cored/** **primitive/immature**	**Cored/classic/dense/** **mature/focal**	- 5–15 µm; - dense core without surrounding neuritic component [65] - visible using silver stain, Congo red [52], and thioflavin [53]
	- 20–60 µm - altered neurites lacking Aβ core in the central part [54] - invisible with Congo red [56]	- 20–60 µm - compact core surrounded by fibrillar deposits of Aβ [46] - tau-positive dystrophic neurites [57], reactive astrocytes, and activated microglia [58,59] in the vicinity - related to neuronal loss and associated with cognitive decline [60,61] - basis of Alzheimer’s disease diagnosis [62] - confirmed with silver stain [63], Congo red [64], and thioflavin [57]	

**Table 2:** Summary of PrP plaque types in transmissible spongiform encephalopathies (TSEs).

PrP Plaques			
- Extracellular Deposits of PrP Visible with Hematoxylin-eosin Staining			
Unicentric/“Kuru”/”Kuru-like”/Stellate	Daisy/Florid	Multicentric	Neuritic
- up to 30 µm in Creutzfeldt–Jakob disease (CJD), 20–60 µm in kuru disease - consisting of a dense star-shaped core and thin amyloid bundles radiating into the periphery [156] - astrocytic processes located in the vicinity - invaded by microglia [157] - present in patients carrying the MV2 polymorphism [138] at codon 129 (total of 10–15% sCJD) [156] - visible with hematoxylin-eosin [90]	- up to 200 µm [132] - composed of PrP-amyloid core surrounded by a “ring” of spongiform changes - thick fibrils radiating into the periphery - tau-positive dystrophic neurites are present [140,159] - visible with hematoxylin-eosin [162]	- up to 1500 µm - composed of many cores of different sizes that have merged together - presence of tau-positive dystrophic neurites [140] - visible with hematoxylin-eosin staining [164]	- clusters of dystrophic neurites that do not contain amyloid structures - surrounded by astrocytic processes [156]
